# Ameliorating Iron Overload in Intestinal Tissue of Adult Male Rats: Quercetin vs Deferoxamine

**DOI:** 10.1155/2018/8023840

**Published:** 2018-11-21

**Authors:** Arwa A. El-Sheikh, Shimaa Hamed Ameen, Samaa Salah AbdEl-Fatah

**Affiliations:** ^1^Forensic Medicine and Clinical Toxicology Department, Faculty of Medicine, Zagazig University, Egypt; ^2^Antomy and Embryology Department, Faculty of Medicine, Zagazig University, Egypt

## Abstract

**Objective:**

The aim of our study is to compare the role of the new natural alternative (Quercetin) with the current iron-chelation therapy (Deferoxamine (DFO)) in the effect of iron overload on small intestinal tissues and to investigate the possible underlying molecular mechanisms of such toxicity.

**Methods:**

Forty-two adult male albino rats were divided into six groups: control groups, DFO, Quercetin, iron overload, iron overload+DFO, and iron overload+Quercetin groups. Animals received daily intraperitoneal injection of Deferoxamine (125 mg /kg), Quercetin (10 mg/kg), and ferric dextran (200 mg/kg) for 2 weeks.

**Results:**

Iron overloaded group showed significant increase in serum iron, total iron binding capacity (TIBC), transferrin saturation percentage (TS %) hepcidin (HEPC), serum ferritin, nontransferrin bound iron (NTBI), and small intestinal tissues iron levels. Iron overload significantly increased the serum oxidative stress indicator (MDA) and reduced serum total antioxidant capacity (TAC). On the other hand, iron overload increased IL6 and reduced IL10 in small intestinal tissues reflecting inflammatory condition and increased caspase 3 reactivity indicating apoptosis and increased iNOs expressing cell indicting oxidative stress especially in ileum. In addition, it induced small intestinal tissues pathological alterations. The treatment with Quercetin showed nonsignificant differences as compared to treatment with DFO that chelated the serum and tissue iron and improved the oxidative stress and reduced tissue IL6 and increased IL10 and decreased caspase 3 and iNOs expressing cells in small intestinal tissues. Moreover, it ameliorated the iron overload induced pathological alterations.

**Conclusion:**

Our study showed the potential role of Quercetin as iron chelator like DFO in case of iron overload induced small intestinal toxicity in adult rats because of its serum and tissue iron chelation, improvement of serum, and small intestinal oxidative stress, ameliorating iron induced intestinal inflammation, apoptosis, and histopathological alterations.

## 1. Introduction

Iron is essential element in various physiological processes in the body as erythropoiesis, oxidative energy production, mitochondrial respiration, and oxygen transport [1].

Although the iron is important metal for the function of cells, however its excess accumulation causes iron overload that may result from hereditary hemochromatosis and related disorders, secondary iron overload as Ineffective erythropoiesis, and excess oral or parenteral iron overload [2].

The iron metabolism is regulated by many factors such as hepcidin, which is peptide hormone that affects iron absorption in small intestine and systemic iron regulation [3]; transferrin (Tf) that bind iron at physiological iron concentrations and nontransferrin bound iron (NTBI) in iron overload concentrations [4]; serum ferritin (SF) and Hemosiderin that reflect the total body iron stores and act as indicative of iron overload [1].

Iron overload produce reactive oxygen species (ROS) leading to impairment of cellular function and multiple disorders as anemia, heart failure, liver cirrhosis, kidney injury, fibrosis, diabetes, arthritis, depression, impotency, infertility, and cancer [5]. Iron induced toxicity increased infection, inflammation, and tissue iron deposition [6].

Iron is absorbed from duodenum which is the main area where iron absorption takes place. However, it is less absorbed in the jejunum and ileum [7]. Thus, the intestinal epithelium is the first barrier against absorption of iron.

Iron removal by chelation therapy is considered important line of treatment strategy in iron overload and toxicity conditions. There are synthetic chelators as deferoxamine due to antioxidant abilities suggesting its use; however some side effects were reported with its use as ototoxicity and ocular toxicity as well as growth retardation [8].

In addition, unreactive ferrioxamine is formed; transferrin-bound iron and heme iron are resistant to chelation by deferoxamine; however, nontransferrin-bound iron and the intracellular labile iron pool are available for chelation [9].

Quercetin is one of natural dietary flavonoids that are present in foods including vegetables, fruit, and tea. It produces protection against various diseases as osteoporosis, certain forms of cancer, pulmonary and cardiovascular diseases, and aging [10]. It prevents cell death by scavenging oxygen radicals, protects against lipid peroxidation [11], and chelates metals [12].

The present work aimed to investigate the ameliorating and chelating effect of Quercetin, compared with the common iron chelator deforoxamine on certain biochemical iron indices, immunohistchemical and histopathological alterations induced by iron overload toxicity on small intestine in adult albino rats. Moreover, it investigated the possible underlying molecular mechanisms of such toxicity.

## 2. Material and Methods

### 2.1. Chemicals

All used chemicals were of the highest quality available. Reagent kits used for measuring rat serum iron, total iron binding capacity (TIBC) and rat hepcidin (HEPC) were obtained from My-Biosource, Inc. USA. Reagents used for detection of serum ferritin were obtained from lifespan biosciences, Inc., USA. Reagent kits used for determination of rat Interleukin-6(IL-6) and Interleukin-10(IL-10) were manufactured by Thermo Fisher Scientific, Inc., UK. Reagent kits used for determination of malondialdehyde (MDA) and total antioxidant capacity (TAC) were purchased from Bio-diagnostic Co., Egypt. All other reagents used for determination of Non transferrin bound iron (NTBI) and tissue iron level were obtained from Sigma-Aldrich (Merck Darmstadt), Germany.

### 2.2. Drug and Treatments

The elemental iron was in form of ferric dextran provided as Fercayl ampules produced by Sunny pharmaceutical, Egypt. Each ampoule of 2 ml solution contains elemental iron (100 mg).

Deferoxamine Mesylate (DFO) was provided as desferal vials obtained from Novartis pharma, Egypt. Each vial contains 500 mg dry white powder dissolved in saline.

Quercetin (Q) was purchased from Sigma Chemical Company, St. Louis, MO, USA. It was provided as yellow powder (≥98% HPLC) dissolved in saline.

### 2.3. Animals

Forty-two male albino rats were obtained from Animal House of Faculty of Medicine, Zagazig University, with an average body weight of 160-200gm. The rats were housed in well-ventilated plastic cages. All animals had free access to balanced laboratory diet and water ad libitum. Animals were acclimated to the housing conditions (12-h light/dark cycle, temperature 25°C, and relative humidity (40%–60%)) for one week before experiment. All animal procedures were performed in accordance with the Guidelines for Ethical Conduct for the care and use of laboratory animals [13].

### 2.4. Experimental Design

The animals were divided into 6 groups. Group I (control group) consisted of 12 rats which were equally subdivided into (negative control) where rats received only regular diet and tap water and (positive control) and rats received normal saline 0.9% (diluent of Iron dextran and Quercetin) (1 ml/kg/day) for 2 weeks.

The other groups consisted of 6 rats per each, where in Group II (Deferoxamine (DFO)), rats received daily intraperitoneal injection of deferoxamine (125 mg/kg) for 2 weeks [14]. In Group III (Quercetin group), rats received daily intraperitoneal injection of Quercetin (10 mg/kg) for 2 weeks [15]. In Group IV (Iron overload group), rats received daily intraperitoneal injection of ferric dextran (200 mg/kg) for 2 weeks [16]. In Group V (Iron overload &DFO treated group), rats received ferric dextran and DFO (30 min after iron administration) as the same doses, the same route, and for the same duration mentioned in two previous corresponding groups. In Group VI (Iron overload and Quercetin treated group), rats received ferric dextran and Quercetin (30 min after iron administration) as the same doses, the same route, and for the same duration mentioned in two previous corresponding groups.

### 2.5. Sample Collection and Tissue Preparation

Twenty-four hours after the last dose of each treatment, the rats (had been fasted over-night) and venous blood samples were collected from retro-orbital plexus of each rat while the animal was anesthetized with ether. The blood samples were centrifuged for obtaining of serum for different biochemical analysis. The animals were then euthanized by cervical dislocation and small intestines were excised. The small intestine of each rat was washed with distilled water and portions of duodenum, jejunum, and ileum of each rat were stored at–80°C for iron and Interleukins assay. The other portions from duodenum, jejunum, and ileum of each rat were washed with saline for several times and were fixed in 10% neutral buffered formalin for histopathological and immunohistochemical examinations. The biochemical analyses were performed in the Department of Biochemistry and Central Laboratory, Faculty of Veterinary Medicine, Zagazig University.

### 2.6. Serum Iron Indices Assay

Serum samples were obtained for estimation of serum iron levels (*μ*mol/L) according to method of [17]. Serum TIBC (*μ*mol/L) was estimated according to instructions of the manufacturer using sandwich enzyme-linked immunoassay method (ELISA), and the serum transferrin saturation (TF %) that determined the transferrin bound iron and its availability to tissues was calculated from TF% = (Serum iron/TIBC) ×100. HEPC (ng/ml) and Ferritin (ng/ml) were estimated according to instructions of the manufacturer using ELISA. NTBI (*μ*mol/L) that represents iron not bound to transferrin and not corresponding to heme and ferritin iron was estimated according to method described by [18] using HPLC technique.

### 2.7. Serum Oxidative Stress and Total Antioxidant Capacity Assay

Serum samples were obtained for estimation of serum Malondialdehyde accumulation and total antioxidant capacity (TAC) according to instructions of the manufacturer using colorimetric method. The data was expressed as mM/L and nmol/ml, respectively.

### 2.8. Small Intestine Iron Levels Assay

Portions of duodenum, jejunum, and ilium of each rat's small intestine were undergone wet digestion by acid mixture (3 ml Nitric acid: 2 ml Perchloric acid), and the dissolving remaining metal matter and the resultant solution were aspirated in to Atomic Absorption Spectrophotometer (AAS) (Buck scientific 210VGP Atomic Absorption Spectrophotometer) for determination of iron level according to [19] and Perkin Elmer model (spectra-AA10, USA) flame atomic absorption spectrometer with computer system was employed. The data was expressed as mg/g wet weight (ppm).

### 2.9. IL-6 and IL-10 Assay in Small Intestinal Tissues

The levels of IL-6 and IL-10 in duodenum, jejunum, and ilium of each rat's small intestine homogenate were determined according to instructions of the manufacturer using ELISA. The small intestinal tissues were rinsed in iced saline, dried, and weighted then underdone centrifugation in low temperature leaving supernatant. The data was expressed as pg/ml.

### 2.10. Histopathological Examination

Paraffin-embedded intestinal tissues were cut in 5 micron thick sections and were stained with haematoxylin and eosin [20].

### 2.11. Immunohistochemical Examination

Immunohistochemistry was performed using primary antibodies against Caspase 3 (apopain, SCA-1, Yama, and CPP32 US Biological, USA) and anti-inducible nitric oxide synthase (anti-iNOS) (isoform Nitros Oxide) (dilution 1: 50; Santa Cruz Biotechnology) using streptavidin–biotin immunoperoxidase technique (DakoCytomation, California, USA). Formalin-fixed, paraffin-embedded (FFPE) tissues were cut into (3-4 *μ*m) thick sections and transferred to 3aminopropyltriethoxysilane (APTS) coated glass slides. Then, sections were subjected to dewaxing, rehydration, blocking with hydrogen peroxide, and antigen retrieval that was performed by heating specimens at 100°C for 20 min in citrate buffer (PH 6.0) within microwave. One to two drops of the primary ready-to-use monoclonal antibody, Caspase 3, and anti-iNOS were then placed on the sections on separate slides. Slides were incubated at room temperature for 60 min. Incubation with secondary antibody and product visualization (Dako) was performed with DAB chromogen (3,3-diaminobenzidine tetrahydrochloride). Sections were counterstained with hematoxylin, dehydrated with ethanol and xylene, and mounted permanently with Din-butylPhthalate in Xylene (DPX). Assessment of Cytoplasmic immunostaining for iNOS was done according to [21] while assessment of brown reaction product in antigen-containing cells with blue stained background [22].

### 2.12. Statistical Analysis

The data were analyzed using the statistical package program SPSS version 23 Software for Windows. The results were expressed as mean ± SE of studied groups using the analysis of variance test (one-way ANOVA) followed by post hoc tests using LSD to make multiple comparisons between all studied groups.

## 3. Results

### 3.1. Effects of Quercetin as Compared to Deferoxamine on Serum Iron Indices in Iron Overloaded Rats

The mean values of serum iron indices revealed nonsignificant differences between both of DFO and Quercetin groups as compared to control groups (P > 0.05). All serum iron indices showed highly significant increase in rats of iron overload group as compared to control groups (P< 0.001).

Treatment with deferoxamine in (iron + DFO group) and Quercetin in (Iron + Quercetin group) showed highly significant reduction in all iron indices as compared to Iron overload groups (P< 0.001). Regarding the effect of Quercetin in (Iron + Quercetin group) as compared to DFO in (iron + DFO group), it showed nonsignificant differences (P > 0.05) in serum iron level, HEPC, ferritin, and NTBI and significant differences in TIBC and TS% (P < 0.05) with preference of Quercetin Table 1.

### 3.2. Effects of Quercetin as Compared to Deferoxamine on Small Intestinal Iron Levels in Iron Overloaded Rats

The mean values of iron levels in all parts of small intestine revealed nonsignificant differences between both DFO and Quercetin groups as compared to control groups (P > 0.05). The results showed significant increase of iron levels in all parts of small intestine, especially in ilium in iron overload group when compared to control groups (P<0.001).

These levels showed significant reduction on administration of DFO in (iron + DFO group) and Quercetin in (iron+ Quercetin group) when compared to iron overload group (P< 0.001). Regarding the effect of Quercetin with iron overload, it showed nonsignificant differences as compared to DFO with iron overload approximating its chelating effect of iron in small intestinal tissues (P > 0.05) Table 2.

### 3.3. Effects of Quercetin as Compared to Deferoxamine on Serum Oxidative Stress and Total Antioxidant Capacity in Iron Overloaded Rats

The mean values of MDA as a marker of oxidative stress and TAC as an index of endogenous antioxidant defense system revealed nonsignificant differences between both of DFO and Quercetin groups as compared to control groups (P > 0.05).

Iron overload induced highly significant increase in MDA and reduction in TAC as indicative of oxidative stress. While on examination of both deferoxamine and Quercetin treatment effects on iron overload, it was showed that DFO and Quercetin normalized the MDA and TAC levels as compared to iron overload group (P< 0.001). Regarding the effect of Quercetin, it showed nonsignificant differences as compared to DFO in iron+ DFO group (P > 0.05) Table 3.

### 3.4. Effects of Quercetin as Compared to Deferoxamine on IL-6 and IL-10 Assay in Small Intestinal Tissues in Iron Overloaded Rats

The mean values of IL6 and IL10 assay in all parts of small intestine revealed non-significant differences between both of DFO and Quercetin groups as compared to control groups (P > 0.05). The results indicated significant increase in IL6 and significant reduction in IL10 in all parts of small intestine of iron overloaded group with more affection of the ileum when compared to control groups (P<0.001).

These results were normalized on treatment with DFO in (iron + DFO group) and Quercetin in (Quercetin+ iron group) when compared to iron overloaded group (P<0.001). Regarding the effect of Quercetin, it showed nonsignificant differences as compared to DFO in their corresponding groups with iron overload. The effects of DFO and Quercetin were noticed in all parts of small intestine with preference of Quercetin in results of IL10 (P > 0.05) Table 4.

### 3.5. Histopathological Results

**Figure 1:**
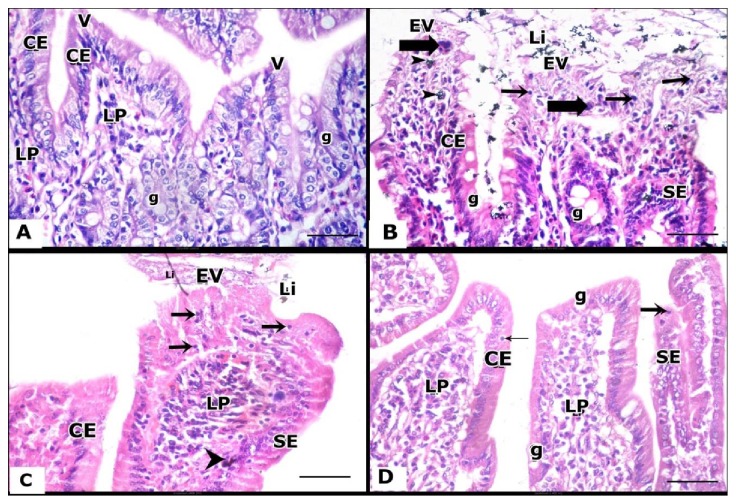
Representative photographs of the histologic sections of intestinal duodenal tissues obtained from the studied groups.** A:** The duodenal tissues of control group show normal histological architecture, while villi appear as a finger like projections with pointed ends** (V)** lined by a single layer of columnar epithelium with basal oval nuclei (**CE)**, few goblet cells** (g),** and lamina propria (**LP**) with few cellular elements can be seen.** B:** The duodenal tissues of iron-overloaded group. The villi show loss of epithelial lining, abundant intraepithelial lymphocytes (**arrow**), iron deposit (arrow head), and giant cells (**thick arrow**) at their tops. Numerous goblet cells are also observed (**g**). Villi are also showing area of single epithelial columnar layer (**CE**) and areas of stratified epithelial lining (**SE**). Luminal iron (**Li**) overeroded villi (**EV**) are seen.** C:** Duodenal tissues of iron +DFO treated group show an eroded villus (**EV**) with area of single epithelial columnar layer (**CE**) and another part shows stratified epithelial lining (**SE**). Prominent lamina propria (**LP**) with iron deposit (**arrow head**) and demarcated intraepithelial lymphocytes (**arrow**) are clarified. Luminal iron (**Li**) is noticed.** D:** Duodenal tissues of iron + Quercetin-treated group show villi with area of single columnar layer with basal oval nuclei (**CE**) and small part shows stratified epithelial lining (**SE**). Prominent lamina propria (**LP**) with demarcated intraepithelial lymphocytes (**arrow**) and goblet cell (**g**) are clarified.** (H&E × 400).**

Examination of H&E stained small intestinal sections of each of controls, DFO, and Quercetin groups showed the same normal histological architecture of small intestine that comprises 4 distinct layers; the mucosa, submucosa, muscularis externa, and serosa. The mucosa consisted of 3 layers, including the epithelium, lamina propria, and muscularis mucosae, and is organized into villi. In the duodenum, villi were finger-like projections with pointed clear ends protruded into the intestinal lumen and lined by a single layer of columnar epithelium with basal oval nuclei and few goblet cells. The underlying lamina propria contained few cellular elements. The submucosa contained mucosal gland and pale stained Brunner's glands. Muscularis externa was also identified (Figure 1(A)).

**Figure 2:**
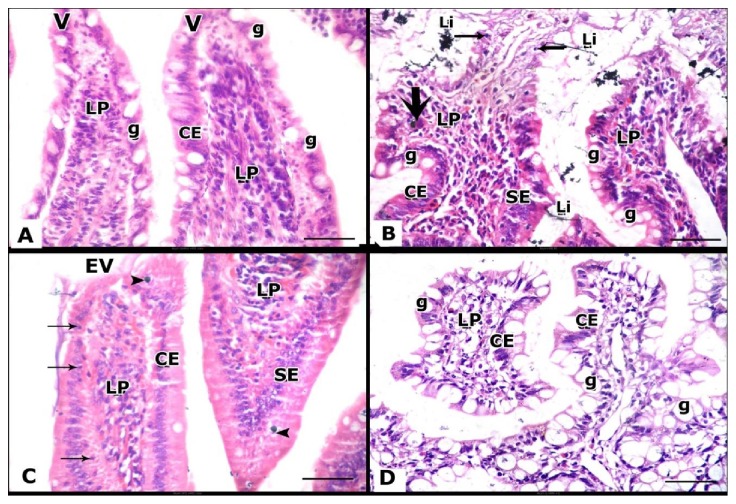
Representative photographs of the histologic sections of ileal tissues obtained from the studied groups.** A:** The ileal parts from control group show normal histological architecture, while villi appear widely separated villi (**V**) and lined by columnar cell with basal oval nuclei (**CE**), numerous goblet cells (**g**) and lamina propria (**LP**) with abundant cellular elements can be seen.** B**: The ileal tissues of ironoverload group show eroded villi with epithelial lining loss, numerous goblet cells (**g**), areas with single columnar layer (**CE**), and areas with stratified epithelial lining (**SE**) and intraepithelial lymphocytes (**thin arrow**) are observed. Lamina propria (**LP**) with giant iron laden cell (**thick arrow**) are clarified. Luminal iron (**Li**) in crystalline and linear forms is observed.** C:** The ileal tissues of iron + DFO treated group show an eroded villus (**EV**). Villi with areas with single columnar layer (**CE**) are areas with stratified epithelial lining (**SE**) are seen. Lamina propria (**LP**), intraepithelial lymphocytes (**arrow**) and iron deposit in epithelium or lamina propria (**arrow head**) are observed.** D**: ileal tissues of iron+ Quercetin-treated group show villi with numerous goblet cells (**g**), while single columnar layer (**CE**) and lamina propria (**LP**) are clarified**. (H&E × 400).**

The jejunum and ileum are histologically identical; the findings were more prominent in ileum than jejunum so the ileum was chosen for studying the treatments effects. In ileum, widely separated villi lined with a single layer of columnar epithelium with basal oval nuclei and numerous goblet cells. Lamina propria with abundant cellular elements could be seen (Figure 2(A)).

In iron overload group, the H&E stained small intestinal sections showed distortion of mucosal architecture. In duodenum, iron deposits were observed in the lumen, epithelium and also at villi tops. The lining epithelium was lost in some villi. Some villi showed areas of single epithelial columnar layer and others showed hyperplasia of columnar epithelium (stratified epithelial lining) and numerous goblet cells. Abundant intraepithelial lymphocytes and also giant cells were also identified at villi epithelial lining. Iron laden cells and demarcated intraepithelial lymphocytes were observed in the lamina propria (Figure 1(B)).

While, the ileum showed luminal iron deposited in different forms as crystalline and linear forms. Nearly most of ileal villi were absent. The reaming eroded villi showed lost epithelial lining, numerous goblet cells, areas with single columnar layer, areas with hyperplasia (stratified epithelial lining), and intraepithelial lymphocytes. Giant iron laden cells were observed within lamina propria (Figure 2(B)).

Moreover, by examination of H&E stained small intestinal sections obtained from iron and deferoxamine received rats showed slight improvement as compared to iron overloaded group. In duodenum, little eroded villi showed areas of single epithelial columnar layer, and others showed hyperplasia (stratified epithelial lining). Demarcated intraepithelial lymphocytes and iron deposit in prominent lamina propria were still clarified. Less luminal iron was noticed (Figure 1(C)).

While in Ileum, the mucosa still showed somewhat of eroded villi. Luminal iron and exfoliated villi were also observed. Intraepithelial lymphocytes and iron deposit were still observed in lamina propria (Figure 2(C)).

However, examination of H&E stained small intestinal sections of Iron and Quercetin treated group showed more improvement of histological architecture of small intestine. In duodenum, apparently normal villi with blunt ends except small part showed stratified epithelial lining. Prominent lamina propria appeared with less demarcated intraepithelial lymphocytes (Figure 1(D)).

While the ileum showed more improvement in its architecture when compared with iron and deferoxamine treated rats. Villi with a single layer of epithelium and numerous goblet cells and lamina propria were identified. No epithelial hyperplasia could be identified (Figure 2(D)).

### 3.6. Immunohistochemical Results

**Figure 3:**
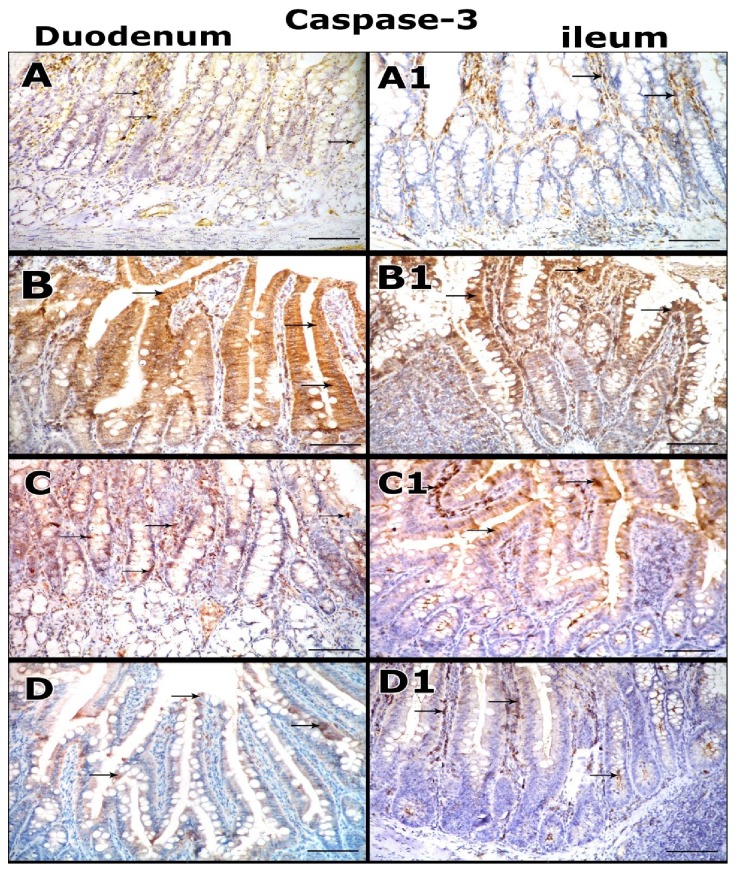
Immunohistochemical staining micrographs of intestinal tissues (duodenum and ileum) of the studied groups for Caspase 3 reactivity (**arrow**).** A, A1:** Control group section shows few weak Caspase-positive cells.** B, B1:** iron overload group section shows numerous strongly positive cells.** C, C1:** Iron +DFO group section shows decrease of caspase-3 reactivity.** D, D1:** Iron+ Quercetin group section shows that marked reduction of the Caspase reactivity in most cells or few strong positive cells is detected** (immunoperoxidase, haematoxylin counter-stain x200).**

Regarding the immunohistochemical staining by caspase-3, the examination of small intestinal tissues sections taken from control, DFO and Quercetin groups showed weak proapoptotic caspase-3staining in both duodenum and ileum (Figure 3(A) and (A1), respectively).

While, the examination of intestinal tissues sections of iron overloaded group showed strong proapoptotic caspase-3 in both duodenum and ileum (Figure 3(B) and (B1), respectively).

However, the immunohistochemical examination of intestinal tissues sections of iron and DFO received group showed less strong proapoptotic caspase-3 staining in both duodenum and ileum (Figure 3(C) and (C1). respectively).

Moreover, the examination of intestinal tissues sections of iron and Quercetin group showed markedly reduction of caspase 3 reactivity in most cells of both duodenum and ileum (Figure 3(D) and (D1), respectively).

**Figure 4:**
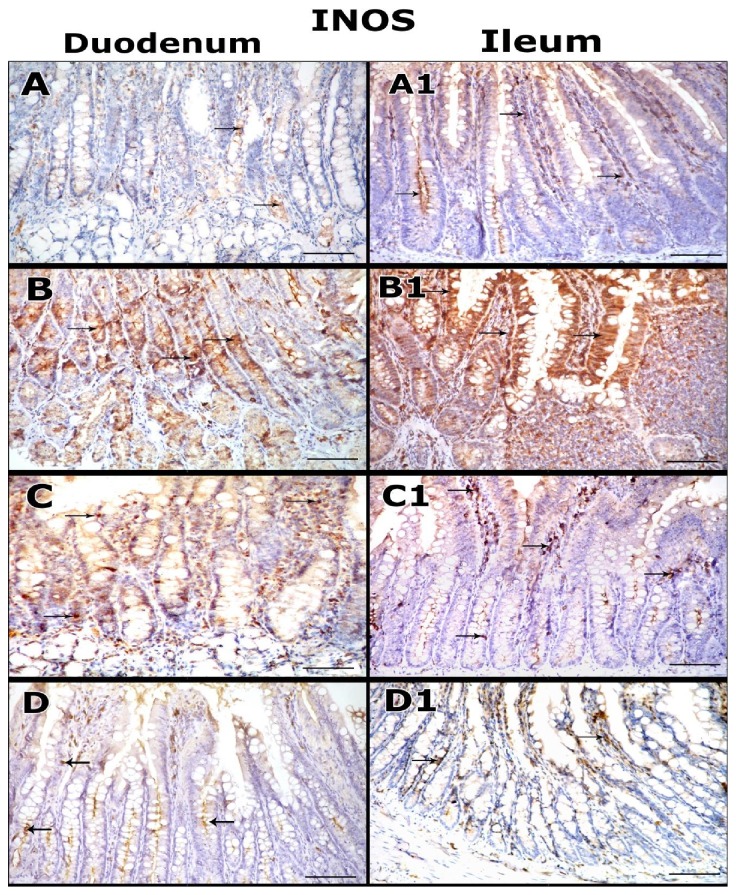
Immunohistochemical staining micrographs of intestinal tissues (duodenum and ileum) of the studied groups for iNOS expressing cells (**arrow**).** A, A1:** Control group section shows few weak iNOS-positive cells.** B, B1:** Iron overload group section shows numerous strongly positive cells.** C, C1:** Iron + DFO group section shows reduction of iNOS expressing cells.** D, D1:** Iron+ Quercetin group shows marked reduction of the expressing cells in most areas** (immunoperoxidase, haematoxylin counter-stain, x200).**

According to the immunohistochemical expressions of inducible nitric oxide synthase (iNOS), the examination of small intestinal tissues sections taken from control, DFO and Quercetin groups showed few weak positive iNOS-expressing cells and in both duodenum and ileum (Figure 4(A) and (A1), respectively).

However, the examination of intestinal tissues sections of iron overloaded group showed numerous strong positive iNOS expressing cells in both duodenum and ileum (Figure 4(B) and (B1), respectively).

However, the immunohistochemical examination of intestinal tissues sections of iron and DFO received group showed reduction of iNOS expressing cells in both duodenum and ileum (Figure 4(C) and (C1), respectively).

Moreover, the examination of intestinal tissues sections of iron and Quercetin group showed markedly reduction of iNOS expression in most cells of duodenal and ileal tissues (Figure 4(D) and (D1), respectively).

## 4. Discussion

Iron overload that caused by excess iron intake led to tissues damage. Although DFO is effective as iron chelator, however it was recorded that it produced major side effects as retinal and auditory toxicity and increased further risk of Yersinia infection with DFO treatment [23]. In addition, DFO affects growth and bone especially if given before puberty and produces localized reactions at prolonged infusion sites [9]. Moreover, other iron chelators have major side effects [24]. These side effects of medications available for treatment of iron overload motivate us to seek pharmacologically alternatives that present in nature and in functional foods that possess antioxidative activities. Quercetin is a typical flavonoid present in propolis and healthy foods such as fruits and vegetables, especially onions, broccoli, apples, and tea [25].

In the present study, the iron toxicity was evident by significant elevation of serum iron, serum TIBC, TS%, NTBI, serum ferritin and HEPC. In addition, the treatment of iron overload group by both of iron chelator (DFO) and Quercetin supplementation separately showed reduction of serum iron, small intestinal iron concentration (duodenum, jejunum and ilium), serum TIBC, TS%, NTBI, serum ferritin, and HEPC. Regarding the Quercetin supplementation effect as compared to DFO, it was shown that both treatments have the same effects on all iron indices. However, the effect of Quercetin in amelioration of TIBC and TS% was more than that of DFO.

Our results of serum iron overload indices were in agreement with several studies recorded different models of iron overload [26–29]. The toxicity may be attributed to elevation of NTBI in iron overloaded rats that was formed when iron binding capacity of transferrin was saturated. NTBI which is toxic than transferrin-bound iron and distributed throughout the organs independent on transferrin receptors [30] catalyzed the formation of reactive radicals [31]. Although, serum ferritin amount is very low; however it is affected by infections, acute and chronic inflammation [1]. Hepcidin (peptide hormone) acts as negative regulator for iron release from cells [32] by binding to ferroportin producing its internalization and degradation [33]. Its circulating levels are affected by the body iron state where it increased in iron overload conditions [34].

The chelation by DFO was introduced 50 years ago, and still has the principle role in treatment of acute and chronic iron overload. DFO is a hexadentate iron chelator that form water soluble stable complex with iron (ferrioxamine) and has large molecular weight, not orally absorbed and excreted renally [35]. Previously, several studies investigated the possible protective role of Quercetin. Lesjak et al. [36] described reduction of serum iron and transferrin saturation levels after Quercetin administration in iron overloaded rats that given iron for 2 weeks. Periyasamy et al. [37] reported that Quercetin protected liver, heart and kidney from doxorubicin induced toxicity in rats. Chan et al. [15] reported that administration of Quercetin by different routes (intraperitoneal and oral) prevented trichostatin A induced DNA damage and lipid peroxidation. Another study showed its protective action on alteration of genes expression and oxidative stress induced by aflatoxin in hepatic tissues of rats [38].

Moreover, our study demonstrated elevation of small intestinal mucosal iron concentrations (duodenum, jejunum and ilium) with more elevation in duodenum and ilium mucosa. Treatment iron overload group with DFO and Quercetin showed marked reduction of iron levels in all parts of small intestine emphasizing the potentiality of Quercetin as a chleator.

These results are consistent with [36] who demonstrated increased iron duodenal level in rats after iron administration for 2 weeks. Little information is available about the effect of iron overload, DFO and Quercetin on intestinal mucosa, especially, regarding levels of iron concentrations. However, in our study, Quercetin played a role in chelation of iron from circulatory pool and intestinal tissues; this may be attributed to what was proven by Lesjak et al. [35] who argued that Quercetin affected intestinal iron absorption through chelation of iron within the intestinal lumen via 3-hydroxyl group of its structure in acute iron overload and by direct regulation of Ferroportin transporter expression in long term iron overload. Moreover, it was suggested that Quercetin supplementation increased iron excretion [30] via formation of Quercetin-iron complex that shuttled through glucose transporter 1 decreasing intracellular hydroxyl radical formation [39].

In the current study, the elevation of iron indices was associated with oxidative stress condition by increased serum MDA level and reduction of serum total antioxidant capacity. Moreover, the treatment of iron overload group by both of iron chelator (DFO) and Quercetin supplementation separately showed reduction of serum MDA and normalization of serum TAC.

These results are in agreement with previous studies of iron overload inducing oxidative stress [26, 27, 40]. The oxidative stress may result from formation of highly reactive hydroxyl radicals from hydrogen peroxide or by producing reactive perferryl and ferryl ions by excess iron [41] leading to sever eloss of total antioxidant status level [42].

Regarding the effect of DFO on oxidative stress induced by iron overload, several studies demonstrated its ameliorating role in attenuating the oxidative stress induced by iron overload in different models [43–45]. DFO action was suggested due to its ability to inhibit the oxidative stress induced by excess iron and reduce ferryl myoglobin which is oxidized myoglobin aggregates that produce oxidative damage [46]. Moreover, other studies have proved the Quercetin ability to improve the oxidative stress induced by iron overload [47, 48]. The role of Quercetin in inhibition of oxidative stress was attributed to its interference with inducible nitric-oxide synthase activity (iNOS); iNOS produced nitric oxide that react with free radicals producing the highly damaging peroxynitrite that caused irreversible damage to the cell membrane [49, 50].

In the current study, there were increased IL6 and reduction of IL10 in small intestinal mucosal tissues of iron overloaded rats indicating increased intestinal mucosal inflammatory response; these findings are consistent with Yi-chen et al. [28] who reported increased levels of IL-6 and reduction of IL10 in intestinal tissue of high-ferrous sulfate administrated rats; another study found increased duodenal mucosal cytokines expression on administration of high iron diet in porcine models [51]. The intestinal inflammatory response associated with iron overload may be attributed to oxidative stress induced by excess cellular iron that increased intestinal mucosal permeability leading to antigen and pathogen translocation though epithelial cells [52]. Meanwhile, Hepcidin released in response to inflammatory cytokines, especially interleukin-6 [53].

While the treatment of iron overload group by both of iron chelator (DFO) and Quercetin supplementation separately showed reduction of IL6 and normalization of IL10 in small intestinal mucosal tissues (duodenum, jejunum, and ilium) in both groups with better effects was shown with Quercetin treatment on IL10, no studies have been conducted on the treatment effects of DFO or Quercetin on the iron overload induced intestinal mucosal inflammation. However, a pervious study was done on the effect of Quercetin on colitis induced in rats; it was found that Quercetin caused rapid inhibition of free radical generation that may reduce the leukocyte infiltration into the inflamed tissue, preventing intestinal tissues from inflammation [54]. The inflammatory response reduction of Quercetin may be attributed to its ability to reduce tumor necrosis factor-*α* (TNF-*α*) and interleukin-1*β* (IL-1*β*) expression from macrophage leading to prevention of systemic inflammation [55].

The current study demonstrated that iron caused several pathological alterations including distortion of mucosal architecture, iron deposits in the lumen, epithelium and villi tops. Some of intestinal villi were eroded and others were atrophied, the columnar epithelium showed hyperplasia, and also numerous goblet cells, Iron laden cells, and demarcated intraepithelial lymphocytes were observed in the lamina propria in duodenum sections and absent most of villi in the ileum. These results were in agreement with [28] who reported that high iron dose in rats caused irregular villous morphology and necrotic intestinal mucosal epithelium, suggesting that excessive iron caused intestinal inflammatory injury in rats. The pathological alterations of small intestine induced by iron were attributed to affection of intestinal hemostasis either by altering intestinal microbial formation, or by oxidative stress, or by inflammation [56, 57].

However, administration of DFO caused slight improvement in small intestinal tissues when compared to iron overloaded rats, as it reduced the luminal iron deposits however, there were still some atrophied and eroded villi in both duodenum and ilium.

These results were consistent with the findings of Yatmark et al. [45] who reported that short term therapy by DFO (2 weeks) resulted in reduction of iron accumulation and improved the pathological changes in the lungs of iron-overloaded mice.

While administration of Quercetin caused more improvement of histological architecture of small intestine as compared to iron overload & iron+DFO received groups, in the form of disappearance of iron deposits, normally apparent villi, No epithelial hyperplasia could be identified in both duodenum and ilium.

No studies have been conducted on the treatment effects of Quercetin on the iron overload induced intestinal mucosal damage. However, a study demonstrated that Quercetin chelate iron similarly active to deferoxamine, because of antioxidative properties of Quercetin [58] that discussed before in addition to its potential role in reduction of inflammatory response.

The immunhistochemical studies of intestinal tissues sections of iron overload group showed strong proapoptotic caspase-3and numerous strong positive iNOS expressing cells in both duodenum and ileum. While, administration of DFO in iron + DFO group showed less strong proapoptotic caspase-3reactivity and reduction of iNOS expressing cells in most cells of both duodenum and ileum. However, the intestinal tissues sections of iron + Quercetin showed marked reduction of Caspase 3 reactivity and iNOS expression in most cells of duodenal and ileal tissues indicating the better effects of Quercetin in decreasing the proapoptotic and oxidative stress activity of iron on the intestinal tissues directly than that of DFO.

The previous studies demonstrated the proapoptotic activities of iron overload through caspase-3 in the mucosal cells of gastrointestinal tract in rats [59] and in preconfluent cells of intestinal cell culture [60]. The oxidative stress activity of iron overload through iNOS expression was studied before on rat liver that reported upregulation of iNOS expression and attributed this expression to generation of reactive oxygen species [61].

Within the experimental studies that have been carried out to date, we are unable to identify a study simultaneously investigating the effects of DFO or Quercetin on apoptosis and iNOS expression in small intestinal tissues induced by iron overload. However, a pervious study recorded the ability of Quercetin to reduce the apoptotic activity in intestinal mucosa induced by methotrexate [62]. Another study reported the capability of Quercetin to inhibit the up regulated colonic iNOS activity in colitic rats and attributed its intestinal role to anti-inflammatory effect and antioxidant or scavenging properties [54].

## 5. Conclusion

The current study suggested that Quercetin may represent a new horizon in treatment of iron overload induced small intestinal toxicity like deferoxamine, via chelation of serum and intestinal iron accumulation, improving serum iron indices (TIBC, TS, HEPC, Ferritin, and NIBT), inhibiting oxidative stress, and improving the antioxidant capacity. It attenuated iron induced apoptotic activity and inflammation. In addition, it ameliorated the histopathological alterations induced by iron overload in small intestinal tissues of the adult rats.

## Figures and Tables

**Table 1:** Effects of Quercetin in comparison to DFO on serum iron indices in iron overloaded rats.

**Groups**	**Serum iron** **(** ***μ*** **mol/L)**	**TIBC** **(** ***μ*** **mol/L)**	**TS**%	**HEPC** **(ng/ml)**	**Ferritin** **(ng/ml)**	**NTBI** **(*μ*mol/L)**
**Control**	18.50±0.640	38.52±0.168	48.02±1.474	4.97±0.07	24.87±0.52	1.83±0.140
**DFO**	18.40±0.590	38.83±0.293	47.82±1.259	4.71±0.08	23.61±0.54	1.51 ±0.257
**Quercetin**	18.25±0.602	38.17±0.333	47.42±1.265	4.99±0.08	23.61±0.55	1.54±0.076
**Iron**	69.17±3.16** a**	73.43±2.68**a**	92.48±2.091 **a**	26.41±0.24a	61.83±1.45**a**	20.67±1.78**a**
**Iron + DFO **	21.53±1.25**b**	40.00±0.516**b**	53.89±2.335 **b**	10.59±0.38**b**	30.24±0.51**b**	2.05±0.292**b**
**Iron+Quercetin **	20.80± 0.957**b,c**	39.51±0.541**b,d**	52.64±2.321**b,d**	10.49±0.25**b,c**	32.31±1.37**b,c**	2.06±0.856**b,c**

**P-Value**	0.001	0.001	0.001	0.001	0.001	0.001

**LSD 0.05**	3.5301	3.120	4.9601	0.624	2.651	1.7504

Values are expressed as means ±standard error (n=6), LSD: least significance difference DFO: Deferoxamine, TIBC: total iron binding capacity, HEPC: hepcidin, and NTBI: nontransferrin bound iron.

a: highly significant difference as compared to control group (P< 0.001).

b: highly significant difference as compared to iron overload group (P< 0.001).

c: non-significant difference as compared to iron + DFO group (P > 0.05).

d: significant difference as compared to iron + DFO group (P < 0.05).

**Table 2:** Effects of Quercetin in comparison to DFO on small intestinal iron levels in iron overloaded rats.

**Groups**	**Duodenum iron level(mg/g wet weight) ± SE**	**Jejunum iron level(mg/g wet weight) ± SE**	**Ilieum iron level (mg/g wet weight) ± SE**
**Control**	0.055±0.0005	0.0361±0.0001	0.0267±0.0002
**DFO**	0.0533±0.0005	0.0359±0.0000	0.0266±0.0001
**Quercetin**	0.0551±0.0004	0.0361±0.0001	0.0268±0.0001
**Iron**	0.0881±0.0009 **a**	0.0563±0.0004** a**	0.1348±0.0002** a**
**Iron + DFO **	0.0611±0.0005** b**	0.0365±0.0001 **b**	0.0282±0.0003** b**
**Iron+Quercetin **	0.0617±0.0004 **b,c**	0.0369±0.0002** b,c**	0.0287±0.0005 **b,c**

**P-Value**	0.001	0.001	0.001

**LSD 0.05**	0.0016	5.478 × 10^−4^	7.164 × 10^−4^

Values are expressed as means ±standard error (n=6), LSD: least significance difference, DFO: deferoxamine.

a: highly significant difference as compared to control group (P< 0.001).

b: highly significant difference as compared to iron overload group (P< 0.001).

c: nonsignificant difference as compared to iron + DFO group (P > 0.05).

**Table 3:** Effects of Quercetin in comparison to DFO on serum oxidative stress and total antioxidant capacity in iron overloaded rats.

**Groups**	**MDA** **(mM/L) ± SE**	**TAC** **(nmol/ml) ± SE**
**Control**	10.86 ± 0.09	1.34 ± 0.02
**DFO**	10.50 ± 0.15	1.36 ± 0.01
**Quercetin**	10.44 ± 0.14	1.37 ± 0.01
**Iron**	34.2 ± 0.59 **a**	0.53 ± 0.01 **a**
**Iron + DFO **	12.74 ± 0.59 **b**	1.31 ± 0.05 **b**
**Iron+Quercetin **	11.77 ± 0.74 **b,c**	1.27 ± 0.04 **b,c**

**P-Value**	0.001	0.001

**LSD 0.05**	1.3356	0.0804

Values are expressed as means ± standard error (n=6), LSD: least significance difference, DFO: Deferoxamine, MDA: malonyldialdehyde, and TAC: total antioxidant capacity.

a: highly significant difference as compared to control group (P< 0.001).

b: highly significant difference as compared to iron overload group (P< 0.001).

c: non- significant difference as compared to iron + DFO group (P > 0.05).

**Table 4:** Effects of Quercetin in comparison to DFO on IL6 and IL10 of small intestinal tissues in iron overloaded rats.

**Groups**	**IL6(pg/ml)**			**IL10(pg/ml)**		
	**Duodenum**	**Jejunum**	**Ilium**	**Duodenum**	**Jejunum**	**Ilium**
**Control**	30.31±0.28	30.24±0.26	29.78±0.25	13.57±0.57	13.65±0.55	13.45±0.044
**DFO**	29.87±0.24	29.88±0.26	29.66±0.24	13.37±0.59	13.34±0.57	13.33 ±0.051
**Quercetin**	29.46±0.16	29.44±0.18	29.44±0.16	12.89±0.54	12.80±0.55	12.55±0.45
**Iron**	88.86±0.57 **a**	94.76±0.61** a**	99.89±0.57** a**	7.43±0.16 **a**	7.01±0.14** a**	6.34±0.11** a**
**Iron + DFO **	47.99±1.64 **b**	44.57±1.43** b**	47.34±1.6** b**	13.04±0.42 **b**	12.89±0.44** b**	12.79±0.53** b**
**Iron+Quercetin **	48.91±1.79 **b,c**	45.67±1.77**b,c**	48.24±1.79**b,c**	12.49±0.18 **b,c**	12.77±0.14**b,c**	12.69±0.11**b,c**

**P-Value**	0.001	0.001	0.001	0.001	0.001	0.001

**LSD 0.05**	2.9612	2.933	2.876	1.2821	1.2835	1.3215

Values are expressed as means ±standard error (n=6), LSD: least significance difference, DFO: deferoxamine,

a: highly significant difference as compared to control group (P< 0.001).

b: highly significant difference as compared to iron overload group (P< 0.001).

c: nonsignificant difference as compared to iron + DFO group (P > 0.05).

## Data Availability

The statistical analysis data and images used to support the findings of this study are included within the article, while the detailed values and separate images are available from the corresponding author on reasonable request.
